# Socioeconomic and ethnic disparities in major lower limb amputation related to peripheral arterial disease in England

**DOI:** 10.1093/bjsopen/zraf046

**Published:** 2025-06-03

**Authors:** Thaison Tong, Ravi Maheswaran, Jonathan Michaels, Paul Brindley, Stephen Walters, Shah Nawaz

**Affiliations:** School of Medicine and Population Health, University of Sheffield, Sheffield, UK; School of Medicine and Population Health, University of Sheffield, Sheffield, UK; School of Medicine and Population Health, University of Sheffield, Sheffield, UK; School of Architecture and Landscape, University of Sheffield, Sheffield, UK; School of Medicine and Population Health, University of Sheffield, Sheffield, UK; Sheffield Vascular Institute, Sheffield Teaching Hospitals NHS Foundation Trust, Sheffield, UK

## Abstract

**Background:**

Amputation is a treatment of last resort for peripheral arterial disease. This study examined associations between socioeconomic deprivation, ethnicity, above-knee amputation (AKA) and below-knee amputation (BKA) rates, and post-amputation survival in England.

**Methods:**

Hospital Episode Statistics data identified patients aged ≥25 years who underwent an AKA or BKA related to peripheral arterial disease in 2006-2018. Data on ethnicity, comorbidity and socioeconomic deprivation was recorded. An ecological study design, based on population-level data, analysed amputation rates (Poisson regression), and a cohort study design investigated mortality subsequent to amputation (Cox regression).

**Results:**

Within a population of 35.7 million people aged ≥25 years, 47 249 patients underwent peripheral arterial disease-related major amputation over 12 years (94.1% White, 1.9% Black, and 1.6% Asian ethnicity). AKA : BKA ratios were 1.03, 0.73, and 0.80 for White, Black, and Asian ethnicities respectively. Amputation rates increased with increasing socioeconomic deprivation. The amputation rate ratio for the most relative to the least deprived category varied with age, ranging from 4.94 (95% confidence interval 4.24 to 5.75) for age 45–54 years to 1.35 (1.21 to 1.49) for age ≥85 years for AKA, and from 3.88 (3.44 to 4.37) to 1.12 (0.97 to 1.29) for BKA. Post-amputation mortality hazard ratios also increased with increasing socioeconomic deprivation, ranging from 1.26 (1.04 to 1.53) for age 25–54 years to 1.11 (1.03 to 1.19) for age ≥75 years for AKA, and from 1.25 (1.08 to 1.46) to 1.17 (1.08 to 1.27) for BKA. Over 12 years, amputation rates decreased in all socioeconomic categories in the population aged ≥65 years, but there was little change in the population aged 25–64 years. Black ethnicity was associated with lower adjusted AKA and BKA rate ratios relative to White ethnicity in those aged 25–64 years, and similar AKA but higher BKA rate ratios in those aged ≥65 years. Black ethnicity was also associated with lower post-amputation mortality, except in those aged 25–54 years within 90 days of BKA. Asian ethnicity was associated with lower AKA and BKA rate ratios relative to White ethnicity, but similar post-amputation mortality with some exceptions.

**Conclusion:**

The main policy and practice implications relate to socioeconomic inequalities. Greater efforts are needed in disadvantaged areas to prevent and manage peripheral arterial disease and reduce amputation.

## Teaser

Amputation is a treatment of last resort for peripheral arterial disease (PAD). This study examined associations between socioeconomic deprivation and ethnicity and above-knee (AKA) and below-knee amputation (BKA) rates and post-amputation survival in England. The main results relate to socioeconomic inequalities, with both AKA and BKA rates and mortality following amputation progressively increasing with increasing levels of socioeconomic deprivation. Greater efforts are needed in socioeconomically disadvantaged areas to prevent and manage PAD and to reduce socioeconomic inequalities in amputation rates and mortality after amputation.

## Introduction

Peripheral arterial disease (PAD) can lead to significant health challenges, and the decision to perform a major amputation of the lower limb is typically considered only after other treatment options have been exhausted or have proven ineffective^1^. Amputation rates reported from different countries seem to show significant variation globally^2,3^. Mortality rates following major amputation procedures are notably high^1,4–6^. In response, strategies have been suggested to improve patient outcomes. For example, the Vascular Society for Great Britain and Ireland has issued best practice guidelines^7^, and the UK All-Party Parliamentary Group on Vascular and Venous Disease has called for efforts to address inequalities in amputation rates^8^.

The prevalence of PAD varies along a socioeconomic gradient, with a higher prevalence in populations that are more socioeconomically disadvantaged^9,10^. In addition, the association between socioeconomic disadvantage and the risk of amputation has been documented in several studies internationally^11–17^. However, there is little on the association between socioeconomic disadvantage and survival after amputation^18^.

The association between ethnicity and major amputation has been extensively studied in the USA, with studies using large data sets finding that Black ethnicity is associated with a higher risk of PAD-related major amputation than is White ethnicity^15,16^. In England, studies suggest that Asian ethnicity may lower the risk of PAD-related major amputation compared with White ethnicity^19,20^. However, the risks associated with Black ethnicity in England are unclear, with some studies reporting higher^20^ and others reporting lower^19,21^ risks of amputation associated with Black ethnicity. There appear to be no published studies examining the association between ethnicity and survival after major amputation in England.

The aims of this study were to examine associations between socioeconomic deprivation and ethnicity and population-based PAD-related major lower limb amputation rates and survival after amputation in England.

## Methods

### Hospital Episode Statistics data on hospital admissions and mortality

Management of the National Health Service (NHS) in England is overseen by NHS England. NHS England supplied Hospital Episode Statistics (HES) in-patient data covering admissions to NHS hospitals in England spanning April 2006–March 2018 (data were provided in financial years, which run from 1 April to 31 March the following year), along with linked mortality records. Patients undergoing above-knee amputation (AKA) or below-knee amputation (BKA) were identified using Office of Population Censuses and Surveys Classification of Surgical Operations and Procedures codes, the standard classification system used in the English NHS^22^. Exclusions were made for cases involving traumatic or malignant amputations, which were identified through corresponding International Classification of Diseases, Tenth Revision (ICD-10) codes in the main diagnosis field^23^.

Pseudoanonymized patient identifiers were used to connect all admissions pertaining to each patient. The first admission featuring AKA or BKA was designated as the index admission and the data set was limited to individuals aged ≥25 years.

HES data included information on patient ethnicity under the variable ‘ethnos’. To determine each patient's ethnicity, data from all admission episodes of care were consolidated. In cases where a patient had different ethnicities recorded across episodes, the ethnicity from the most recent episode was retained as the final ethnicity. These ethnicity codes were then grouped into six categories: White, Black, Asian, Mixed, Others, and Missing.

Patients with co-morbidities were identified using ICD-10 codes in the first 14 diagnosis fields in the index admission or in any previous admission within 1 year of the index admission. Eight co-morbidity categories were considered (see results), based on the Royal College of Surgeons Charlson score^24^ and opinions from the expert clinical advisory panel for this project^25^.

### Study design, geography, and socioeconomic deprivation

An ecological study design, based on population-level data, was used to analyse amputation rates. A cohort study design was used to investigate mortality after amputation.

The study used lower layer super output areas (LSOAs) as the basic geographical units, each encompassing an average population of 1500 individuals^26^. Due to minimal alterations to LSOAs between the 2001 Census and the 2011 Census, the analysis focused on 31 672 of the 32 844 LSOAs in 2011 (representing 96.4% of the total) to ensure consistency throughout the study period.

The Index of Multiple Deprivation (IMD) is a nationally recognized index of deprivation and is extensively used by government agencies throughout England^27^. The Income Domain from IMD 2010 was used as the measure of socioeconomic deprivation at the level of LSOAs.

LSOA mid-year population estimates by age and sex from 2006 to 2017 were used to calculate annual amputation rates by socioeconomic deprivation category.

Ethnicity-specific population data by age and sex at the LSOA level were only available for the 2011 census year. Consequently, population rate estimates by ethnicity were confined to using HES data for the five HES years spanning 2011 (April 2009–March 2014) combined. These estimates were used to calculate rates in broad age bands because of the more limited population data set.

### Statistical analysis

Time trends in population-based rates by socioeconomic deprivation category are presented as graphs. Population-based rates in relation to deprivation and ethnicity were examined using Poisson regression. Mortality after amputation in relation to deprivation and ethnicity was initially investigated using Kaplan–Meier survival curves, and associations were analysed using Cox proportional hazards modelling, with follow-up to 31 March 2018. Mortality hazard ratios (HRs) following amputation were estimated in three age bands because there were different patterns in some age bands. In addition, in some ethnicity age bands, associations varied with time and the associations in these age bands were examined in the 0–90-day and >90-day periods. Results are presented as adjusted rate ratios or HRs with 95% confidence intervals (c.i.).

Because data were very sparse at the LSOA level, LSOAs were grouped into five categories using socioeconomic deprivation quintiles, and the median deprivation value within each category was used as a continuous variable in the statistical analyses. Rate ratios and HRs were calculated as a trend across all quintile categories and presented as the ratio (with 95% c.i.) for the most relative to the least deprived category.

## Results

In the 12-year time span analysed, there were 47 249 patients with major lower limb amputation related to PAD, with 50.7% being AKA. This occurred within a population aged ≥25 years of 35.7 million. Consequently, the overall annual rate of PAD-related major lower limb amputation in individuals aged ≥25 years was 11 per 100 000.

The majority of patients (66%) were aged ≥65 years, with men accounting for 67.8% of all patients who underwent major lower limb amputation (*Table 1*). In terms of socioeconomic deprivation, 27.3% of patients were in the most deprived category by quintile and 12.1% were in the least deprived category. With regard to ethnicity, 94.1% of patients were of White ethnicity, 1.9% were of Black ethnicity, and 1.6% were of Asian ethnicity. The remainder were either of Mixed ethnicity (0.3%), Other ethnicity (0.7%), or had missing ethnicity information (1.4%). The overall AKA to BKA ratio was 1.03. The most prevalent co-morbidities were diabetes (48.0%) and coronary heart disease (35.4%) (*Table 1*).

**Table 1:** Characteristics of the 47 249 patients aged ≥25 years who had PAD-related major lower limb amputation overall and according to AKA and BKA (April 2006–March 2018)

Characteristic	All	AKA	BKA
**Age (years)**			
25–34	642 (1.4%)	184 (0.8%)	458 (2.0%)
35–44	2061 (4.4%)	597 (2.5%)	1464 (6.3%)
45–54	4581 (9.7%)	1475 (6.2%)	3106 (13.3%)
55–64	8767 (18.6%)	3704 (15.5%)	5063 (21.7%)
65–74	13 044 (27.6%)	6778 (28.3%)	6266 (26.9%)
75–84	12 841 (27.2%)	7532 (31.5%)	5309 (22.8%)
≥85	5313 (11.2%)	3665 (15.3%)	1648 (7.1%)
All	47 249 (100.0%)	23 935 (100.0%)	23 314 (100.0%)
**Sex**			
Male	32 028 (67.8%)	14 943 (62.4%)	17 085 (73.3%)
Female	15 221 (32.2%)	8992 (37.6%)	6229 (26.7%)
**Deprivation category**			
1 (most deprived)	12 918 (27.3%)	6544 (27.3%)	6374 (27.3%)
2	11 039 (23.4%)	5581 (23.3%)	5458 (23.4%)
3	9666 (20.5%)	4803 (20.1%)	4863 (20.9%)
4	7896 (16.7%)	4076 (17.0%)	3820 (16.4%)
5 (least deprived)	5730 (12.1%)	2931 (12.2%)	2799 (12.0%)
All	47 249 (100.0%)	23 935 (100.0%)	23 314 (100.0%)
**Ethnicity**			
White	44 484 (94.1%)	22 579 (94.3%)	21 905 (94.0%)
Black	882 (1.9%)	371 (1.6%)	511 (2.2%)
Asian	748 (1.6%)	332 (1.4%)	416 (1.8%)
Mixed	144 (0.3%)	75 (0.3%)	69 (0.3%)
Others	319 (0.7%)	158 (0.7%)	161 (0.7%)
Missing	672 (1.4%)	420 (1.8%)	252 (1.1%)
All	47 249 (100.0%)	23 935 (100.0%)	23 314 (100.0%)
**Co-morbidities**			
Coronary artery disease	16 728 (35.4%)	8841 (36.9%)	7887 (33.8%)
Heart failure	9345 (19.8%)	5148 (21.5%)	4197 (18.0%)
Cerebrovascular disease	5666 (12.0%)	3437 (14.4%)	2229 (9.6%)
COPD	10 516 (22.3%)	6035 (25.2%)	4481 (19.2%)
Diabetes	22 702 (48.0%)	8759 (36.6%)	13 943 (59.8%)
Renal disease	10 684 (22.6%)	4962 (20.7%)	5722 (24.5%)
Cancer	3043 (6.4%)	1786 (7.5%)	1257 (5.4%)
Moderate or severe liver disease	482 (1.0%)	260 (1.1%)	222 (1.0%)

AKA, above-knee amputation; BKA, below-knee amputation; COPD, chronic obstructive pulmonary disease.

The AKA to BKA ratios in the five socioeconomic deprivation categories by quintile were broadly similar with no apparent trend across categories; the ratios from the most to least deprived category were 1.03, 1.02, 0.99, 1.07, and 1.05, respectively. The prevalence of diabetes was highest in the most deprived category, and gradually decreased with decreasing levels of deprivation. Diabetes prevalence estimates from the most to the least deprived category were 49.5%, 49.0%, 47.6%, 46.7%, and 45.3%, respectively.

The AKA to BKA ratios were 1.03 for White ethnicity, 0.73 for Black ethnicity, and 0.80 for Asian ethnicity. The prevalence of diabetes was 47.3% for White ethnicity, 67.1% for Black ethnicity, and 77.3% for Asian ethnicity.

### Socioeconomic deprivation and amputation rates

There was a clear association between socioeconomic deprivation and amputation rates for both AKA and BKA, which progressively increased with increasing levels of deprivation. The amputation rate ratio for the most relative to the least deprived category varied with age and ranged from 4.94 (95% c.i. 4.24 to 5.75) for those aged 45–54 years to 1.35 (1.21 to 1.49) for those aged ≥85 years for AKA, and from 3.88 (3.44 to 4.37) to 1.12 (0.97 to 1.29) in the corresponding age groups for BKA (*Table 2*).

**Table 2:** Population-based rates for AKA and BKA by socioeconomic deprivation category and age, and rate ratios (adjusted for sex and year) for the most *relative to* the least deprived category (April 2006–March 2018)

Age (years)	*n*	Amputation rate per 100 000 population per year					Rate ratio
		SDC 1 (most deprived)	SDC 2	SDC 3	SDC 4	SDC 5 (least deprived)	
**AKA**							
25–34	184	0.3	0.2	0.2	0.1	0.1	2.66 (1.90, 3.71)
35–44	597	1.4	0.9	0.5	0.4	0.3	4.38 (3.64, 5.27)
45–54	1475	3.8	2.2	1.6	0.8	0.5	4.94 (4.24, 5.75)
55–64	3704	11.3	7.3	4.5	2.9	2.0	4.60 (4.21, 5.02)
65–74	6778	24.5	16.2	11.2	8.3	5.8	3.59 (3.34, 3.86)
75–84	7532	32.1	25.7	20.9	18.2	14.5	2.07 (1.93, 2.23)
≥85	3665	31.8	26.5	25.4	25.9	22.0	1.35 (1.21, 1.49)
**BKA**							
25–34	458	0.8	0.5	0.6	0.5	0.3	1.99 (1.56, 2.54)
35–44	1464	3.2	2.0	1.6	1.0	0.7	3.58 (3.17, 4.05)
45–54	3106	7.1	4.9	3.5	2.1	1.3	3.88 (3.44, 4.37)
55–64	5063	14.1	9.6	6.6	4.4	3.2	3.73 (3.43, 4.06)
65–74	6266	19.8	14.6	10.9	8.3	6.3	2.81 (2.60, 3.05)
75–84	5309	20.6	17.5	16.4	12.9	10.3	1.85 (1.70, 2.01)
≥85	1648	12.0	12.1	11.9	11.9	10.6	1.12 (0.97, 1.29)

Values in parentheses are 95% confidence intervals. AKA, above-knee amputation; BKA, below-knee amputation; SDC, socioeconomic deprivation category (by quintile).

**Fig. 1:**
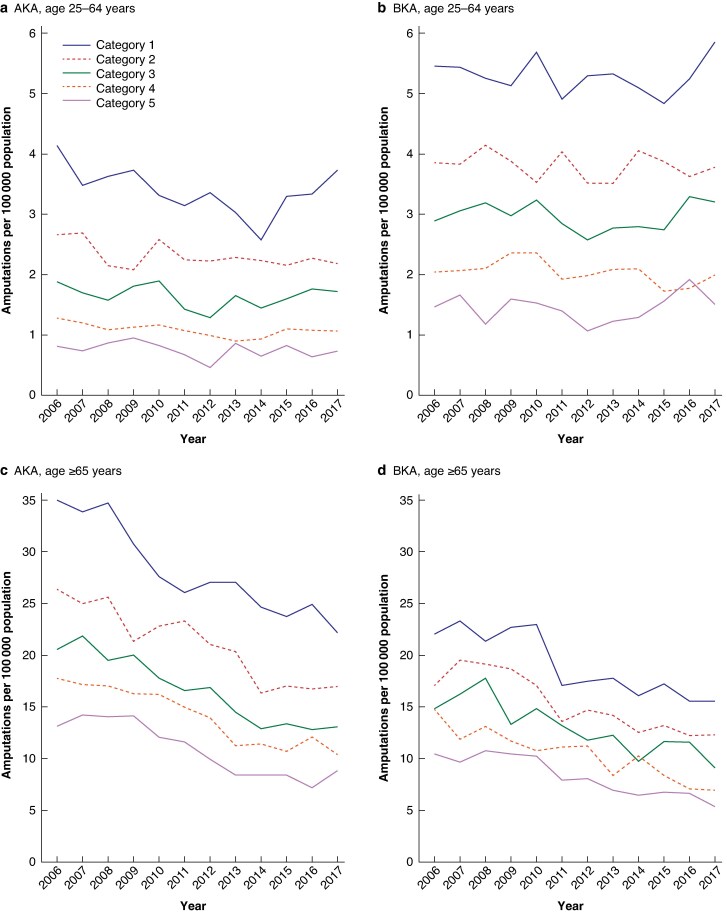
Time trends in AKA and BKA rates by socioeconomic deprivation category and age (April 2006–March 2018) **a** Above-knee amputations (AKA) and **b** below-knee amputations (BKA) in the population aged 25–64 years. **c** AKA and **d** BKA in the population aged ≥65 years.

Over the 12-year study period, both AKA and BKA rates clearly decreased in all socioeconomic categories in the population aged ≥65 years (*Fig. 1*). However, in the population aged 25–64 years, there was generally little change in AKA and BKA rates over the time period examined in all socioeconomic categories.

### Ethnicity and amputation rates

In the population aged 25–64 years, Black ethnicity was associated with lower rates for both AKA and BKA compared with White ethnicity (*Table 3*). The magnitude of the difference was marginally amplified following adjustment for deprivation, with rate ratios adjusted for sex, year, and deprivation of 0.25 (95% c.i. 0.18 to 0.37) for AKA and 0.36 (0.27 to 0.47) for BKA for Black ethnicity relative to White ethnicity.

**Table 3:** Population-based AKA and BKA rates by ethnicity and age, and rate ratios (relative to White ethnicity) before and after adjustment for socioeconomic deprivation (April 2009–March 2014)

Age (years)	Amputation rate per 100 000 population per year		Rate ratio (relative to White ethnicity)	
			Adjusted for sex and year	Adjusted for sex, year and deprivation
	Black ethnicity	White ethnicity		
**AKA**				
25–64	0.8	2.0	0.40 (0.31, 0.52)	0.25 (0.18, 0.37)
≥65	21.3	17.7	1.19 (1.02, 1.40)	0.86 (0.72, 1.02)
**BKA**				
25–64	1.7	3.4	0.53 (0.39, 0.73)	0.36 (0.27, 0.47)
≥65	23.3	13.0	1.77 (1.49, 2.10)	1.31 (1.11, 1.55)
	Asian ethnicity	White ethnicity		
**AKA**				
25–64	0.3	2.0	0.15 (0.11, 0.19)	0.11 (0.07, 0.15)
≥65	7.7	17.7	0.42 (0.36, 0.50)	0.34 (0.29, 0.41)
**BKA**				
25–64	0.8	3.4	0.24 (0.21, 0.28)	0.18 (0.15, 0.22)
≥65	6.6	13.0	0.48 (0.41, 0.57)	0.40 (0.32, 0.50)

Values in parentheses are 95% confidence intervals. AKA, above-knee amputation; BKA, below-knee amputation.

In contrast, in the population aged ≥65 years, Black ethnicity was associated with higher rates of AKA and especially BKA compared with White ethnicity prior to adjustment for deprivation (*Table 3*). However, for AKA, the higher rate was no longer apparent after adjusting for deprivation, with a rate ratio adjusted for sex, year, and deprivation of 0.86 (95% c.i. 0.72 to 1.02). For BKA, the magnitude of the higher rate diminished after adjustment for deprivation but was still apparent, with a rate ratio adjusted for sex, year, and deprivation of 1.31 (95% c.i. 1.11 to 1.55).

Asian ethnicity was associated with noticeably lower amputation rates for both AKA and BKA compared with White ethnicity, and the contrast in rates was especially marked in the population aged 25–64 years (*Table 3*). For AKA, the rate ratio adjusted for sex, year, and deprivation for Asian ethnicity relative to White ethnicity was 0.11 (95% c.i. 0.07 to 0.15) in the population aged 25–64 years, and 0.34 (0.29 to 0.41) in the population aged ≥65 years; for BKA, the corresponding adjusted rate ratios were 0.18 (0.15 to 0.22) and 0.40 (0.32 to 0.5).

### Socioeconomic deprivation and mortality after amputation

The HRs for mortality after amputation, adjusted for age, sex, ethnicity, and year, increased with increasing levels of socioeconomic deprivation (*Table 4*). This general pattern was seen in all three age bands for both AKA and BKA, but the magnitude of the HRs diminished with increasing age. Additional adjustment for co-morbidities made little difference to the HRs. The adjusted HRs for mortality after AKA, including adjustment for diabetes and other co-morbidities, in the most relative to the least socioeconomically deprived category by quintile ranged from 1.26 (95% c.i. 1.04 to 1.53) in the group aged 25–54 years to 1.11 (1.03 to 1.19) in the group aged ≥75 years. The adjusted HRs for mortality after BKA ranged from 1.25 (1.08 to 1.46) in the group aged 25–54 years to 1.17 (1.08 to 1.27) in group aged ≥75 years.

**Table 4:** Adjusted hazard ratios for mortality following AKA or BKA in the most *relative to* the least socioeconomically deprived category by age (April 2006–March 2018)

Age (years)	*n*	Deaths	Hazard ratio	
			Model 1	Model 2
**AKA**				
25–54	2255	823	1.29 (1.07, 1.55)	1.26 (1.04, 1.53)
55–74	10 479	6965	1.22 (1.14, 1.30)	1.19 (1.11, 1.27)
≥75	11 193	9111	1.14 (1.07, 1.22)	1.11 (1.03, 1.19)
**BKA**				
25–54	5025	1495	1.25 (1.09, 1.44)	1.25 (1.08, 1.46)
55–74	11 327	6167	1.25 (1.17, 1.34)	1.23 (1.14, 1.33)
≥75	6954	5386	1.21 (1.12, 1.31)	1.17 (1.08, 1.27)

Values in parentheses are 95% confidence intervals. Model 1 was adjusted for age, sex, ethnicity, and year. Model 2 was adjusted for all the variables in Model 1 and co-morbidities. AKA, above-knee amputation; BKA, below-knee amputation.

### Ethnicity and mortality after amputation

There were more complex patterns in some age bands where the association between ethnicity and mortality after amputation varied during the follow-up period. In these age bands, associations were examined separately in the ≤90-day and >90-day periods after amputation.

For AKA, Black ethnicity was associated with lower mortality HRs, adjusted for age, sex, deprivation, and year, relative to White ethnicity (*Table 5*). Additional adjustment for co-morbidities, including diabetes, did not substantially alter these HRs. The adjusted HRs, including adjustment for co-morbidities, were 0.46 (95% c.i. 0.26 to 0.84) for those aged 25–54 years, 0.65 (0.48 to 0.86) for those aged 55–74 years, and 0.62 (0.50 to 0.76) for those age ≥75 years.

**Table 5:** Adjusted hazard ratios for mortality following AKA or BKA by ethnicity (relative to White ethnicity) and age (April 2006–March 2018)

Age (years)	*n*	Deaths	*n*	Deaths	Hazard ratio (relative to White ethnicity)	
					Model 1	Model 2
	Black ethnicity		White ethnicity			
**AKA**						
25–54	45	13	2088	777	0.67 (0.39, 1.17)	0.46 (0.26, 0.84)
55–74	144	90	9919	6582	0.77 (0.62, 0.95)	0.65 (0.48, 0.86)
≥75	182	123	10 565	8609	0.60 (0.50, 0.73)	0.62 (0.50, 0.76)
**BKA**						
25–54						
0–90 days after procedure	128	14	4662	161	3.25 (1.88, 5.62)	2.50 (1.33, 4.70)
>90 days after procedure	111	23	4415	1240	0.72 (0.48, 1.10)	0.51 (0.32, 0.81)
55–74	224	111	10 640	5797	0.76 (0.63, 0.92)	0.70 (0.55, 0.88)
≥75	159	99	6596	5137	0.54 (0.45, 0.66)	0.51 (0.41, 0.63)
	Asian ethnicity		White ethnicity			
**AKA**						
25–54	52	16	2088	777	0.95 (0.58, 1.56)	0.63 (0.37, 1.07)
55–74	157	106	9919	6582	1.11 (0.91, 1.35)	0.96 (0.74, 1.26)
≥75	122	97	10 565	8609	1.12 (0.91, 1.39)	1.05 (0.82, 1.34)
**BKA**						
25–54	110	38	4662	1401	1.38 (1.00, 1.91)	0.93 (0.62, 1.39)
55–74						
0–90 days after procedure	243	48	10 640	1030	2.25 (1.68, 3.01)	1.81 (1.25, 2.62)
>90 days after procedure	193	92	9437	4767	1.06 (0.86, 1.30)	0.85 (0.67, 1.09)
≥75						
0–90 days after procedure	62	18	6596	1324	1.74 (1.09, 2.77)	1.71 (1.06, 2.76)
>90 days after procedure	44	24	5210	3813	0.61 (0.41, 0.92)	0.56 (0.37, 0.85)

Values in parentheses are 95% confidence intervals. Model 1 was adjusted for age, sex, deprivation, and year. Model 2 was adjusted for all the variables in Model 1 and co-morbidities. AKA, above-knee amputation; BKA, below-knee amputation.

For BKA, although adjusted HRs for mortality after amputation were mostly lower for Black ethnicity relative to White ethnicity, the pattern was more complex in the group aged 25–54 years (*Table 5*). In this age group, Black ethnicity in fact had higher post-amputation mortality within the 90-day period after amputation, with an adjusted HR of 3.25 (95% c.i. 1.88 to 5.62); additional adjustment for co-morbidities reduced this to 2.50 (1.33 to 4.70). However, after the 90-day period, the HR for mortality for Black ethnicity was lower relative to White ethnicity in the group aged 25–54 years, with an adjusted HR, including additional adjustment for co-morbidities, of 0.51 (95% c.i. 0.32 to 0.81). In the groups aged 55–74 and ≥75 years, mortality after BKA was lower for Black ethnicity relative to White ethnicity throughout the follow-up period. The adjusted HRs for BKA, including additional adjustment for co-morbidities, were 0.70 (95% c.i. 0.55–0.88) in the group aged 55–74 years and 0.51 (0.41–0.63) in the group aged ≥75 years.

For Asian ethnicity relative to White ethnicity, mortality after AKA was broadly similar between the two groups (*Table 5*). The adjusted HRs, including adjustment for diabetes and other co-morbidities, were 0.63 (95% c.i. 0.37 to 1.07) for age 25–54 years, 0.96 (0.74 to 1.26) for age 55–74 years, and 1.05 (0.82 to 1.34) for age ≥75 years.

After BKA, in the 25–54 age group, Asian ethnicity had similar mortality to White ethnicity, with an adjusted HR, including adjustment for co-morbidities, of 0.93 (95% c.i. 0.62 to 1.39) (*Table 5*). However, patterns in the 55–74- and ≥75-year age groups were more complex. In both these age groups, mortality within 90 days of BKA was higher for Asian ethnicity relative to White ethnicity. In the group aged 55–74 years, the adjusted HR, including adjustment for co-morbidities, was 1.81 (95% c.i. 1.25 to 2.62), and in the group aged ≥75 years it was 1.71 (1.06 to 2.76).

Beyond the 90-day period after amputation, mortality after BKA for Asian ethnicity in the group aged 55–74 years was similar to that for White ethnicity, with an adjusted HR including adjustment for co-morbidities of 0.85 (95% c.i. 0.67 to 1.09). However, in the group aged ≥75 years, the adjusted HR, including adjustment for co-morbidities, was 0.56 (95% c.i. 0.37 to 0.85), indicating that mortality after the initial 90-day period post-BKA was lower for Asian ethnicity relative to White ethnicity for those aged ≥75 years.

## Discussion

Rates for both AKA and BKA increased progressively with increasing levels of socioeconomic deprivation. Over the 12-year time period examined in this study, amputation rates decreased in all socioeconomic categories in the population aged ≥65 years, but there was little change over time in the population aged 25–64 years. Mortality after amputation increased with increasing levels of socioeconomic deprivation, although the disparity diminished with increasing age.

Black ethnicity was associated with lower population-based adjusted AKA and BKA rate ratios compared with White ethnicity in the population aged 25–64 years, and similar AKA but higher BKA rate ratios in the population aged ≥65 years. Black ethnicity was also associated with lower adjusted HRs for mortality following amputation compared with White ethnicity, the only exception being in the age band of 25–54 years, where it was higher but only within the 90-day period after BKA.

Asian ethnicity was associated with lower population-based adjusted AKA and BKA rate ratios compared with White ethnicity in populations aged 25–64 and ≥65 years. Asian ethnicity had similar adjusted HRs for mortality after amputation compared with White ethnicity. The only exceptions were for people aged ≥55 years, where adjusted HRs for mortality were higher within the 90-day period after BKA and lower in people aged ≥75 years after 90 days post-BKA.

The association between socioeconomic deprivation and major amputation rates found in the present study is consistent with previous studies in a range of countries. A study performed in England and published in 2010 reported an association between deprivation and major lower limb amputation related to PAD^11^. However, that study only used data from the catchment area of a single district general hospital. A study performed in France found that people with diabetic foot ulceration in more deprived areas had a higher risk of major amputation^12^. However, that study was restricted to people with diabetes living in one area of France. A national cohort study in Finland, also restricted to people with diabetes, found that lower household income was associated with a higher risk of major lower limb amputation^13^. National studies in the USA using case-control, cohort and ecological study designs^14–16^, and a meta-analysis of five North American studies^17^, all found associations between deprivation and PAD-related major lower limb amputation.

The present study found that major amputation rates decreased over time in all socioeconomic categories with no clear differences between the categories, but only one previous study, the national cohort study in Finland, appears to have examined trends over time^13^. That study examined major amputation rates between 1993 and 2007 in people with diabetes, classified by quintiles of household income. Although amputation rates decreased in all income groups over time, the relative decrease was greater in less deprived categories, resulting in increased relative inequality over the time period examined.

Mortality after major lower limb amputation increased with increasing levels of socioeconomic deprivation in the present study, but there appears to be little in the published literature on this aspect. The closest is the cohort study in Finland, which examined survival in people with diabetes, using a composite endpoint of major amputation or death, after the first minor amputation^13^. That study found that survival or major amputation-free survival after the first minor amputation was worse in more socioeconomically deprived groups.

In the present study, among the younger and middle-aged population, Black ethnicity was associated with lower AKA and BKA rates compared with White ethnicity. However, in the older population, Black ethnicity was associated with higher BKA rates. Previous UK studies have reported conflicting results, but did not examine how associations varied with age^19–21^. A study using data for England for the period 2003–2009 found that Black ethnicity was associated with higher PAD-related population-based major amputation rates than White ethnicity^20^. In contrast, another study using data for England from 2007 to 2010, which examined the correlation between Primary Care Trust (PCT) level PAD-related major amputation rates and the proportion of Black ethnicity within each PCT, found that PCTs with a higher proportion of Black ethnicity had lower overall major amputation rates^19^. An earlier study using UK data was inconclusive, but it was based on very small counts of incident amputations^21^. However, in the USA, the association is clearer, with Black ethnicity being linked to higher PAD-related major lower limb amputation rates compared with White ethnicity^15,16^.

In the present study, Black ethnicity was generally associated with lower mortality after major amputation compared with White ethnicity, the only exception being in the 25–54-year age band, where mortality was higher but only within the 90-day period after BKA. The generally lower mortality after amputation for patients of Black ethnicity is well documented in studies in the USA. For example, studies using Veterans Affairs health data found that African Americans had a survival advantage after lower limb amputation^28–30^. There appears to be little in the published literature on survival after major amputation in the UK with regard to Black compared with White ethnicity. The apparently high mortality within the 90-day period after BKA for Black relative to White ethnicity in the 25–54-year age band found in the present study warrants further investigation.

In the present study, Asian ethnicity was associated with substantially lower population-based AKA and BKA rates compared to White ethnicity in the population aged ≥65 years and especially in population aged 25–64 years. Previous studies in England found that Asian ethnicity was associated with lower major amputation rates^19,20^. The study which used HES data from 2003 to 2009 found that the risk of major amputation associated with South Asian ethnicity, using ethnicity recorded in the HES, was approximately half that associated with White ethnicity, but it did not distinguish between AKA and BKA^20^. The study that used HES data from 2007 to 2010 analysed at the PCT level essentially performed an ecological (area) level analysis where the proportion of the population in each ethnic category was used in the analysis instead of individual-level ethnicity recorded in HES^19^. That study found that a higher proportion of the PCT-level population with Asian ethnicity was correlated with a lower major amputation rate, but did not distinguish between AKA and BKA^19^.

In the present study, Asian ethnicity was associated with similar mortality to White ethnicity after major amputation. The only exceptions were in people aged ≥55 years, where mortality was higher within the 90-day period after BKA, and lower in people aged ≥75 years beyond 90 days after BKA. There appears to be little in the published literature on survival after major amputation for patients of Asian ethnicity compared to those of White ethnicity.

The higher prevalence of diabetes among patients of Black and Asian ethnicity seen in the present study is consistent with the higher prevalence of diabetes in these ethnic groups in England^31^. The lower AKA:BKA ratios observed for patients of Black and Asian compared with White ethnicity in the present study are likely to be explained by the higher prevalence of diabetes in these two ethnic groups, because AKA:BKA ratios are much lower in patients with than without diabetes^6^.

The rates of major amputation associated with Asian ethnicity were lower relative to White ethnicity in the present study, despite the higher prevalence of diabetes associated with Asian ethnicity. HES data do not contain any reliable information on smoking status. However, a higher proportion of the Asian ethnicity population in England have never smoked regularly compared with the White ethnicity population^32^. This may have partly contributed to a lower prevalence of PAD and major amputation associated with Asian ethnicity despite the higher prevalence of diabetes, although there may also be other complex and less well understood reasons because PAD prevalence is paradoxically low given the high risk of coronary heart disease associated with Asian ethnicity^33,34^.

Black ethnicity was also associated with lower deprivation-adjusted amputation rate ratios relative to White ethnicity in the 25–64-year age group despite the higher overall prevalence of diabetes. In contrast, in group aged ≥65 years, the BKA rate ratio was higher for Black relative to White ethnicity. The proportion of the Black ethnicity population in England who have never smoked regularly, compared with the White ethnicity population, is mixed^32^. A higher proportion of the Black African ethnicity population have never smoked regularly, but for Black Caribbean ethnicity, the proportion of men who have never smoked regularly is lower and similar to that in the White ethnicity male population.

A key strength of HES data is that data capture is likely to be complete because all admissions are likely to be captured by the NHS systems. In addition, HES data are routinely linked to mortality records, allowing reliable estimation of mortality risks. The complete data capture also allows reliable calculation of population-based rates.

However, there are a number of limitations to be considered. There may have been errors in the accuracy of diagnostic and procedure coding in HES, which may have varied over time, leading to misclassification of co-morbidities and operative procedures. Because HES is essentially an administrative data set, it contains very limited clinical information. In addition, LSOA-level population counts are estimates and may not precisely reflect actual population numbers.

The measure of socioeconomic deprivation was only available at the area level rather than at the individual patient level because the NHS system does not collect data on individual socioeconomic status. In addition, using the IMD from a single year may have led to the misclassification of some LSOAs into an incorrect deprivation category, particularly at the extremes of the time period examined.

Coding and interpretation of ethnicity data in HES and of population estimates classified by ethnicity can be problematic. Ethnicity is typically self-reported and can introduce variability because it is subjective, with individuals potentially classifying their ethnicity differently based on context or understanding of the categories provided. This, in addition to data entry errors, may partly explain why some patients had different ethnicities recorded for different admissions. In addition, there may be some mismatch in alignment between coding in HES and census-based population estimates because the data collection methods are different.

There are several potential factors contributing to the differences that were seen in relation to socioeconomic disparities, each of which may have different implications for policy and practice. Such factors include variations in the underlying rate of PAD, delays in seeking medical attention for early symptoms of PAD, and differences in access to, and the provision of, specialist vascular services. The higher incidence of vascular disease in disadvantaged populations may need to be addressed through more targeted measures at the primary care level to provide education and smoking cessation services and to address other risk factors for cardiovascular disease. Delayed presentation among particular populations may be addressed through publicity campaigns and educational activities. Differences in access to specialist services may require changes to service provision, such as the provision of local referral services in areas where centralization may make it more difficult for disadvantaged populations to access remote facilities. Local referral pathways should ensure that patients have equitable access to specialist services, but also ensure that education and awareness of PAD are improved in primary care.

From a policy perspective, more emphasis needs to be given to socioeconomic disparities in vascular policy. The 2022 update of the Best Practice Clinical Care Pathway for PAD for the UK^35^ does not mention socioeconomic inequalities in major amputation rates, and neither does the 2016 Best Practice Clinical Care Pathway for major amputation surgery^7^, but both of these are focused on clinical care, so that is perhaps understandable. However, the National Vascular Registry Healthcare Improvement Strategy for the UK published in 2023 also does not mention socioeconomic inequalities in vascular diseases despite being relevant to a wider audience^36^.

The UK All-Party Parliamentary Group on Vascular and Venous Disease 2019 report entitled ‘Saving limbs, saving lives’ also does not highlight socioeconomic inequalities in major amputations^8^. However, it does highlight ethnic disparities and stated that ‘amputation rates are 70% higher in the Black as compared to the White populations of England. In contrast, the amputation rate in the South Asian population is 40% lower than the White population in England’^8^. The result for BKA in the present study associated with Black ethnicity in the population aged ≥65 years, which found a 77% (95% c.i. 49 to 101%) higher rate ratio before adjustment for deprivation, is consistent with that statement. However, after adjustment for deprivation, the excess amputation risk came down to 31% (95% c.i. 11 to 55%). The equivalent for AKA was a 19% (95% c.i. 2 to 40%) excess risk before adjustment for deprivation, but there was no evidence of excess after adjustment for deprivation (−14%, −28 to 2%).

The Vascular Society Quality Improvement Program includes a recommendation for the AKA:BKA ratio to be <1^37^, and the ratios for Black and Asian ethnicities of 0.73 and 0.80, respectively, found in the present study suggest that, for this quality indicator at least, amputation in relation to Black and Asian ethnicity is not a particular cause for concern. Also of note is that although higher major amputation rates associated with higher levels of socioeconomic deprivation found in the present study are a clear cause for concern, there was little variation in the AKA:BKA ratio across deprivation categories. However, a potential problem with interpretation of the ratio is that it may be distorted as a measure of quality because it may be affected by late presentation and differences in revascularization rates.

Previous work found that there was a clear decrease in revascularization rates for moderate PAD in England following the release of the National Institute for Health and Care Excellence (NICE) guideline for the management of PAD in 2012^38^, with a greater decrease in more socioeconomically deprived areas. In addition, increasing rates of revascularization for severe PAD observed before the guideline plateaued after its release^38^. It is perhaps reassuring to note that there was no clear evidence of an increase in major amputation rates for PAD in the present study as a consequence of the decrease in revascularization rates, with clear progressive decreases in AKA and BKA rates over time observed in all socioeconomic categories in the population aged ≥65 years.

The main implications for policy and practice resulting from the present study relate to socioeconomic inequalities in amputation rates and mortality after amputation. No additional special focus is needed in England in relation to Black and Asian ethnicities regarding PAD-related amputation rates and mortality after amputation, possibly with the exception of the older Black ethnicity population in relation to higher amputation rates for BKA and younger patients of Black ethnicity in relation to higher short-term mortality after BKA. Greater efforts are needed in socioeconomically disadvantaged areas to prevent and manage PAD and to reduce socioeconomic inequalities in amputation rates. This includes improving the provision of and access to NHS stop-smoking services and the early detection and management of PAD, especially chronic limb threatening ischaemia.

## Data Availability

The HES data used in this project may be obtained from NHS England.
